# Long noncoding RNA NEAT1, regulated by LIN28B, promotes cell proliferation and migration through sponging miR-506 in high-grade serous ovarian cancer

**DOI:** 10.1038/s41419-018-0908-z

**Published:** 2018-08-28

**Authors:** Wu Yong, Deng Yu, Zhu Jun, Duan Yachen, Weng Weiwei, Xu Midie, Ju Xingzhu, Wu Xiaohua

**Affiliations:** 1 Department of Gynecologic Oncology, Fudan University Shanghai Cancer Center, Shanghai, China; 2 Department of Oncology, Shanghai Medical College, Fudan University, Shanghai, China; 3 Department of Pathology, Fudan University Shanghai Cancer Center, Shanghai, China

## Abstract

The aberrant expression of long noncoding RNAs (lncRNAs) has been reported frequently in specific cancers, including high-grade serous ovarian cancer (HGSOC). The purpose of the present study was to explore the clinical significance and underlying mechanisms of a significantly dysregulated lncRNA (NEAT1) in HGSOC. Our results showed that elevated NEAT1 expression in human HGSOC specimens correlated with a poor prognosis. Functional experiments demonstrated that knockdown of NEAT1 significantly prohibited ovarian cancer cell proliferation and invasion in vitro and restrained tumor growth in vivo. LIN28B was identified by bioinformatics analysis along with experimental evidence as a direct actor that enhanced NEAT1 stability. A rescue functional assay confirmed that the LIN28B/NEAT1 axis contributed to oncogenic functions in ovarian cancer cells. Moreover, gene expression profile data and dual luciferase reporter assay results demonstrated that NEAT1 functioned as a competing endogenous RNA (ceRNA) for miR-506 to promote cell proliferation and migration. Taken together, our results showed that NEAT1, stabilized by LIN28B, promoted HGSOC progression by sponging miR-506. Thus, NEAT1 can be regarded as a vital diagnostic biomarker for HGSOC and a therapeutic target.

## Introduction

Epithelial ovarian cancer (EOC) is the most lethal gynecological cancer and a common cause of cancer-related death in women worldwide^1,2^. Despite aggressive frontline treatments with surgery and targeted chemotherapy, most patients relapse and die from their disease^2^. High-grade serous ovarian carcinoma (HGSOC) accounts for 60–80% of the women diagnosed with EOC, and most deaths related to EOC are associated with this subtype^3^. Therefore, understanding the pathophysiological mechanisms contributing to HGSOC is of paramount importance for the development of new diagnostic techniques and treatment strategies and the improvement of the overall prognosis of OC patients.

Long noncoding RNAs (lncRNAs), which are a newly discovered class of noncoding RNA (ncRNA) greater than 200 nucleotides in length, have been increasingly reported in a variety of cancer types, suggesting an important role of lncRNAs in human diseases, especially cancer^4,5^. Many studies have demonstrated the diverse cellular functions of lncRNAs, including cell proliferation, cell differentiation, cell apoptosis, and carcinogenesis^5,6^. NEAT1 is an abundant intranuclear lncRNA that contains two transcripts, NEAT1_1 (3.7 kb) and NEAT1_2 (23 kb); the latter transcript is a core component of paraspeckles, which are major complexes involved in RNA nuclear retention that participate in precursor RNA splicing^7–10^. Previous studies have suggested that NEAT1 is an oncogene in various cancers, including lung cancer^11^, hepatocellular cancer^12^, prostate cancer^13^, colorectal cancer^14^, and nasopharyngeal carcinoma^15,16^. Although some studies have revealed that NEAT1 may exhibit malignant biological behaviors in EOC^17^, the detailed mechanisms and functions of NEAT1 in HGSOC have not been clearly elucidated.

Recently, growing knowledge of RNA-binding protein (RBP) targets has directed attention towards ncRNAs, including RNAs involved in translation machinery and its regulation (rRNAs, tRNAs, siRNAs, and miRNAs) as well as the large and heterogeneous class of lncRNAs^18,19^. However, only a small number of lncRNAs have been functionally well characterized to date^20,21^. A few reports have noted that NEAT1 can bind RBPs, such as NONO and PSF^22^. However, relationships between NEAT1 and other RBPs have rarely been reported.

In this study, we found that NEAT1 was overexpressed in HGSOC tissues and that this lncRNA promoted cell proliferation, migration, and invasiveness as well as tumor growth in vivo. Furthermore, mechanistic investigations showed that the upregulation of NEAT1 in HGSOC was mediated by the RBP LIN28B, which bound to and stabilized NEAT1. By determining the downstream effects of NEAT1, our results suggested that the LIN28B/NEAT1 axis might confer an oncogenic function via sponging miR-506. These findings provide new insights into the molecular functions of NEAT1 and shed new light on the treatment of HGSOC.

## Results

### NEAT1 is upregulated in HGSOC and correlates with poor outcomes

**Fig. 1:**
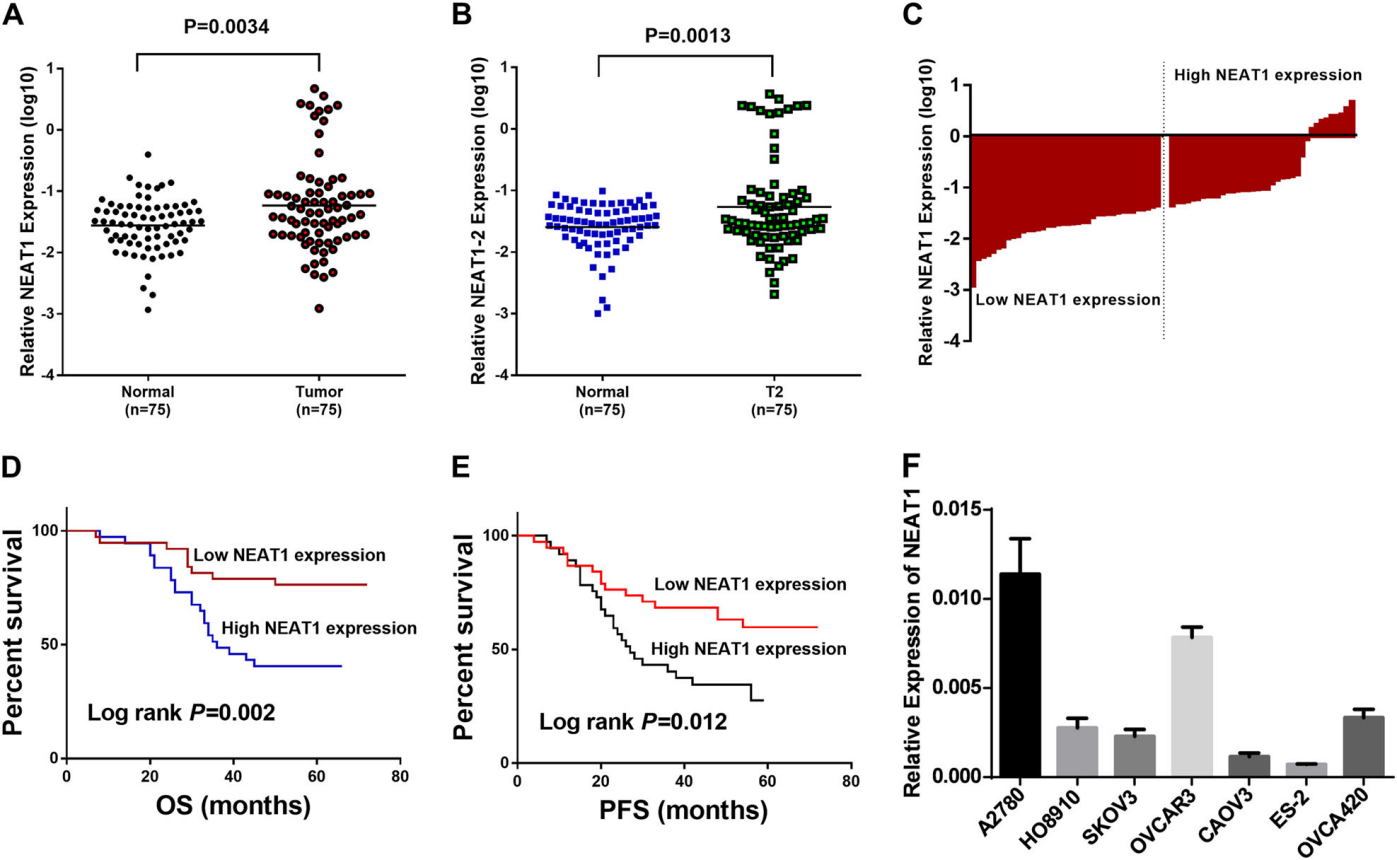
NEAT1 is upregulated in HGSOC and is correlated with patient prognosis. **a** NEAT1 expression levels in HGSOC and unpaired normal ovarian tissues determined using the NEAT1 primer (which can detect both transcripts). **b** NEAT1 expression in HGSOC and normal tissues was determined using the NEAT1-2 primer (which can detect the long transcript). β-actin was used as an endogenous control to normalize the data. **c** A total of 75 HGSOC patients were divided into high- and low-expression groups based on the median expression value. **d** Kaplan–Meier analysis of overall survival in all patients with HGSOC according to NEAT1 expression. **e** Kaplan–Meier curves for PFS of patients based on NEAT1 expression. **f** NEAT1 expression was quantitated in seven EOC cell lines using qRT-PCR

Considering that NEAT1 has two transcripts that share the same 5′ end but are processed alternatively at the 3′ terminus^22^, it was of interest to determine whether one transcript plays a major oncogenic role in HGSOC or the two transcripts have similar roles. To do so, we silenced NEAT1 via an siRNA targeting both NEAT1 transcripts or an siRNA targeting NEAT1-2 only. The two siRNAs resulted in the nearly identical arrest of ovarian cancer cell proliferation and migration (Supplementary Figure S1A, B, C), which suggested that targeting only NEAT1-2, which was recognized as the predominant isoform for the function of NEAT1 in the paraspeckle, did not have a stronger oncogenic effect. Then, we designed two primers named NEAT1 (which can detect both transcripts) and NEAT1-2 (which can detect the long transcript) to assess their expression levels in HGSOC tissues. The qPCR analysis showed that both total NEAT1 and NEAT1-2 were expressed at significantly higher levels in HGSOC tissues than in normal ovarian tissues (Fig. 1a, b; *P* = 0.0034, *P* = 0.0013). Thus, we used the NEAT1 primer in our subsequent experiments unless otherwise specified. Based on the above results, we speculated that the two transcripts share the major oncogenic role in HGSOC. Therefore, siRNAs targeting the common sequences of both transcripts were designed for the subsequent functional experiments.

For the clinicopathological correlation analysis, 75 HGSOC patients were divided into high- and low-expression groups based on the median expression value (Fig. 1c). As shown in Table 1, high NEAT1 expression was closely associated with tumor size (*P*=0.006). Overall survival (OS) and progression-free survival (PFS) curves were plotted according to the NEAT1 expression levels using the Kaplan–Meier method and log-rank test. As shown in Fig. 1d, e, high NEAT1 expression was significantly correlated with both shorter OS (*P* = 0.002) and shorter PFS (*P* = 0.012). The univariate analysis revealed that the relative NEAT1 expression level (*P* = 0.004; *P* = 0.015), International Federation of Gynecology and Obstetrics (FIGO) stage (*P* = 0.016; *P* = 0.036), and vascular invasion (*P* = 0.038; *P* = 0.028) were prognostic indicators of OS (Table 2) and PFS (Table 3). Moreover, the multivariate Cox regression analysis showed that FIGO stage (*P* = 0.011; *P* = 0.006), vascular invasion (*P* = 0.022; *P* = 0.011), and high NEAT1 expression (*P* = 0.007; *P* = 0.026) were independent risk factors of OS (Table 2) and PFS (Table 3). These results suggest that NEAT1 is a potential independent prognostic factor for HGSOC. Furthermore, we determined the NEAT1 expression in the EOC cell lines OVCAR3 and A2780, which were selected for subsequent knockdown experiments because of their high relative expression (Fig. 1f).

**Table 1:** The correlation between NEAT1 and clinicopathological parameters

Variables	*n* (%)	Expression of NEAT1		*χ*^2^	*P* value
		Low	High		
Age (years)				2.259	0.133
≤55	38 (50.6)	23	15		
>55	37 (49.4)	15	22		
Size (cm)				7.469	0.006
≤5	27 (36.0)	13	14		
>5	48 (64.0)	25	23		
FIGO stage				0.108	0.742
I–II	21 (28.0)	10	11		
III–IV	54 (72.0)	28	26		
Poor histologic differentiation				0.015	0.902
Yes	35 (46.7)	18	17		
No	40 (53.3)	20	20		
Vascular invasion				1.382	0.240
Yes	23 (30.7)	14	9		
No	52 (69.3)	24	28		
Lymphatic metastasis				3.316	0.069
Yes	28 (37.3)	18	10		
No	47 (62.7)	20	27		
Distant metastasis				1.139	0.286
Yes	16 (21.3)	10	6		
No	59 (78.7)	28	31

**Table 2:** Univariate and multivariate analysis of clinicopathological factors for overall survival

Variables	Univariate analysis			Multivariate analysis		
	HR	95% CI	*P* value	HR	95% CI	*P* value
NEAT1 expression	3.315	1.439–6.828	0.004*	2.915	1.333–6.377	0.007*
FIGO stage (I–II, III–IV)	3.088	1.079–8.833	0.016*	3.953	1.369–11.413	0.011*
Vascular invasion (Yes, No)	0.362	0.139–0.943	0.038*	0.322	0.122–0.849	0.022*
Lymphatic metastasis	0.586	0.270–1.273	0.177			
Age (≤55, >55)	1.014	0.501–2.053	0.969			
Size (≤5 cm, >5 cm)	0.569	0.280–1.155	0.119			
Poor histologic differentiation (Yes, No)	0.911	0.450–1.843	0.796			
Distant metastasis (Yes, No)	1.070	0.461–2.483	0.875			

*HR* hazard ratio

*P < 0.05

**Table 3:** Univariate and multivariate analysis of clinicopathological factors for progression-free survival

Variables	Univariate analysis			Multivariate analysis		
	HR	95% CI	*P* value	HR	95% CI	*P* value
NEAT1 expression	2.234	1.170–4.266	0.015*	2.096	1.092–4.023	0.026*
FIGO stage (I–II, III–IV)	2.399	1.059–5.432	0.036*	3.194	1.390–7.337	0.006*
Vascular invasion (Yes, No)	0.418	0.192–0.911	0.028*	0.356	0.160–0.789	0.011*
Lymphatic metastasis	0.660	0.340–1.282	0.220			
Age (≤55, >55)	0.840	0.452–1.564	0.583			
Size (≤5 cm, >5 cm)	0.844	0.445–1.603	0.605			
Poor histologic differentiation (Yes, No)	1.167	0.623–2.185	0.630			
Distant metastasis (Yes, No)	1.119	0.533–2.352	0.766			

*HR* hazard ratio

*P < 0.05

### Attenuation of NEAT1 expression inhibits cell proliferation and invasion

**Fig. 2:**
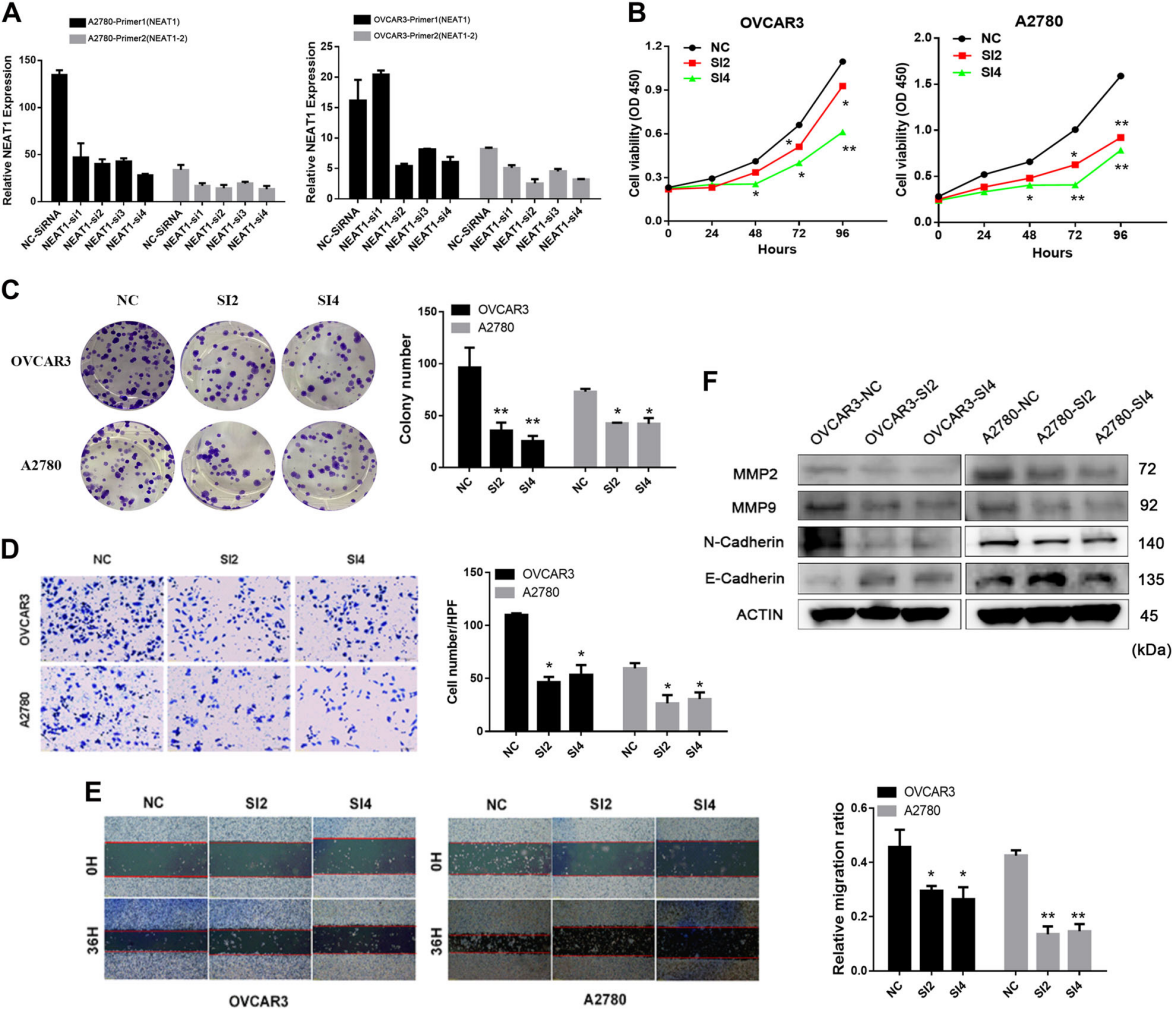
Attenuation of NEAT1 expression inhibits cell proliferation, invasion, and migration in vitro. **a** The EOC cell lines OVCAR3 and A2780 were transfected with siRNAs, and the efficiency of knockdown was verified by qRT-PCR using two NEAT1 primers. **b** CCK-8 assays were performed to determine the proliferation of NEAT1-knockdown cells. **c** The cell colony formation ability of OVCAR3 and A2780 cells was assessed to determine the effects of NEAT1 knockdown on cell growth. **d** The cell invasion potential of the OVCAR3 and A2780 cells was assessed using a Transwell assay. Scale bar is 50 μm. **e** The cell migration ability was evaluated using a wound-healing assay; images of OVCAR3 and A2780 cells were taken at 0 and 36 h postscratch. Scale bar is 200 μm. **f** Western blotting analysis was used to determine the MMP2, MMP9, N-cadherin, and E-cadherin expression levels. Actin was used as a reference. The data are shown as the mean ± SD of three replicates; ^*^P < 0.05, ^**^P < 0.01 vs. NC

To verify that NEAT1 expression was positively associated with HGSOC progression, we first employed four siRNA oligonucleotides to test the knockdown efficiency for this lncRNA in OVCAR3 and A2780 cells. The qPCR results (Fig. 2a) showed that NEAT1-SI2 and NEAT-SI4 significantly reduced both total NEAT1 and NEAT1-2 endogenous expression. Therefore, we chose these two siRNAs to perform the functional experiments. We utilized the CCK8 and colony formation assays to elucidate the potential effect of NEAT1 on cell proliferation. The CCK8 assay showed that cell viability was significantly restrained after NEAT1 downregulation (*P* < 0.05; Fig. 2b). Consistently, the colony formation assays showed that knockdown of NEAT1 led to the formation of fewer colonies in both the OVCAR3 and A2780 cells than in the control cells without NEAT1 knockdown (*P* < 0.05; Fig. 2c). These data suggest that knockdown of NEAT1 represses tumor cell proliferation.

Because cell invasion is an important aspect of cancer progression that involves the migration of tumor cells into contiguous tissues and the dissolution of extracellular matrix proteins, we evaluated the effects of NEAT1 on cell migration and invasion. The Transwell assay results shown in Fig. 2d demonstrated that transfection of the siRNAs targeting NEAT1 impeded the migratory ability of the cells (*P* < 0.05). In addition, the wound assay showed that the ovarian cancer cells transfected with the siRNAs had lower migration capacities than did the control cells (Fig. 2e). Next, we evaluated the expression of epithelial–mesenchymal transition (EMT)-associated proteins and matrix metalloproteinases (MMPs). Western blotting analysis revealed that the epithelial marker gene E-cadherin was significantly increased in the NEAT1-knockdown group relative to the control group (Fig. 2f), whereas the mesenchymal marker N-cadherin was significantly decreased (Fig. 2f). In addition, MMP9 and MMP2 were downregulated in the NEAT1-knockdown group (Fig. 2f). These findings suggested that NEAT1 influenced the migration and invasion of EOC cells.

### NEAT1 interacts with the RBP LIN28B in tumor cells

**Fig. 3:**
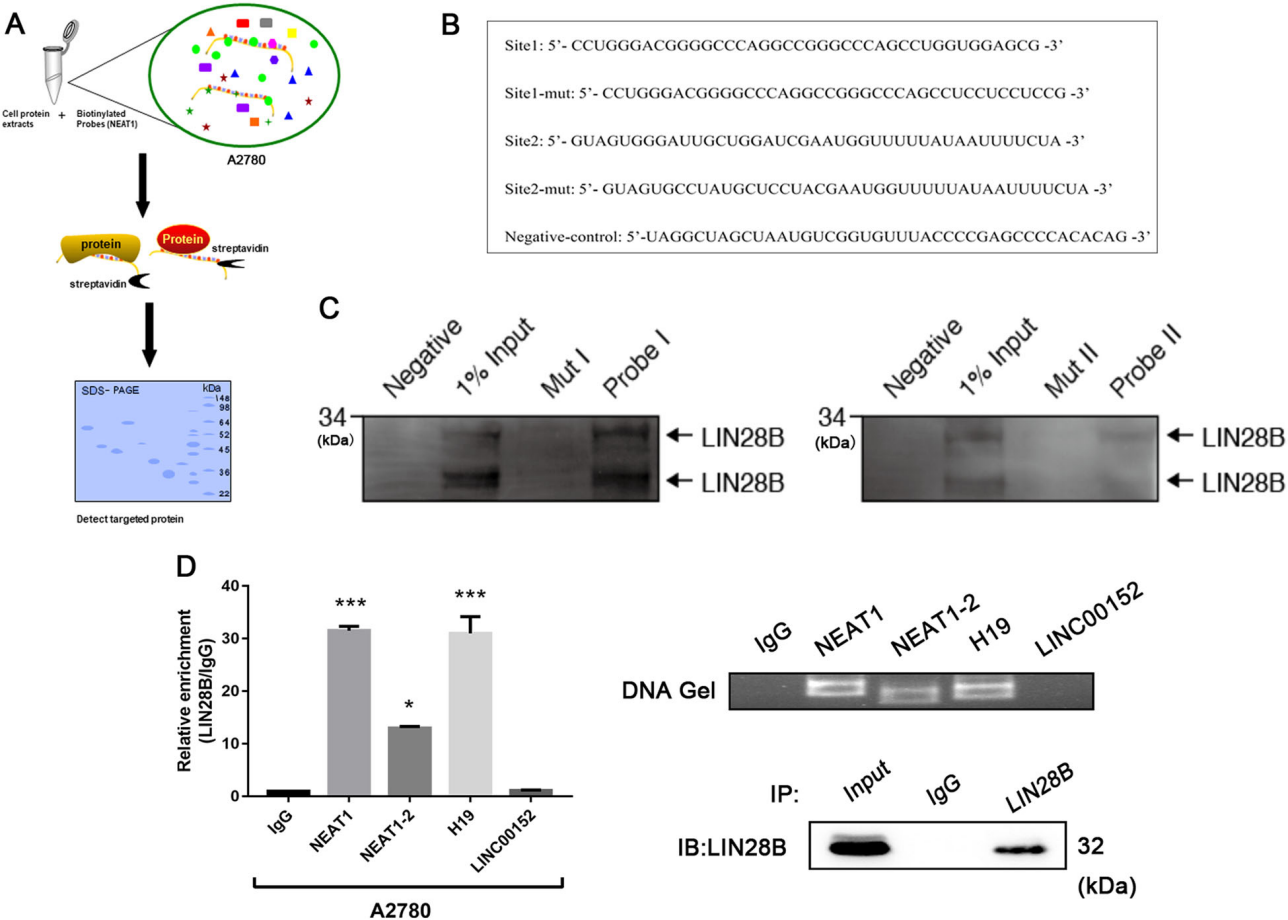
NEAT1 interacts with the RBP LIN28B. **a** A schematic of the RNA pulldown experiment for the identification of proteins associated with LIN28B. **b** Two creditable NEAT1 binding sites were chosen to design the RNA probes and the corresponding mutated probes. **c** RNA pulldown assays using the two groups of biotinylated probes and the mutated probes indicated that NEAT1 interacted with LIN28B. **d** RIP assays showing the association of LIN28B with NEAT1 in A2780 cells. ^*^P < 0.05, ^***^P < 0.001

To explore the molecular mechanism underlying the oncogenic activity of NEAT1 in ovarian carcinogenesis, StarBase V2.0 (http://starbase.sysu.edu.cn/mirLncRNA.php) and RNA-Protein Interaction Prediction (http://pridb.gdcb.iastate.edu/RPISeq/) were used to predict the interaction probabilities of NEAT1 and RBPs. The bioinformatics analysis revealed that NEAT1 could potentially bind to LIN28B. To investigate the potential interaction between the lncRNA NEAT1 and LIN28B, RNA pulldown assays (Fig. 3a) were conducted with the A2780 cell line. In view of the length of NEAT1, we first explored the possible binding sites through CLIP data using the ENCODE database (https://www.encodeproject.org/experiments/ENCSR861GYE/); detailed binding information is provided in Supplementary Table 1. Then, two credible binding sites were chosen to design RNA probes and corresponding mutant probes (Fig. 3b). Subsequently, we confirmed the interaction between NEAT1 and LIN28B through RNA pulldown and Western blotting assays (Fig. 3c). We also performed RNA immunoprecipitation followed by detection of NEAT1 enrichment using qPCR and found that NEAT1 was enriched in the LIN28B samples relative to the IgG group (Fig. 3d). As expected, obvious enrichment of H19 was observed in the LIN28B group (Fig. 3d), as has been reported previously^23^. In contrast, LINC00152, which was regarded as the negative control group, could not bind LIN28B (Fig. 3d). Notably, NEAT1-2 has a weak binding capacity with LIN28B. We suspect that these two transcripts may have different secondary structures; the long isoform mainly binds RBPs, such as NONO and PSF^22^, which may influence the interaction between NEAT1-2 and LIN28B.

### LIN28B enhances the stability of NEAT1 in EOC cells

**Fig. 4:**
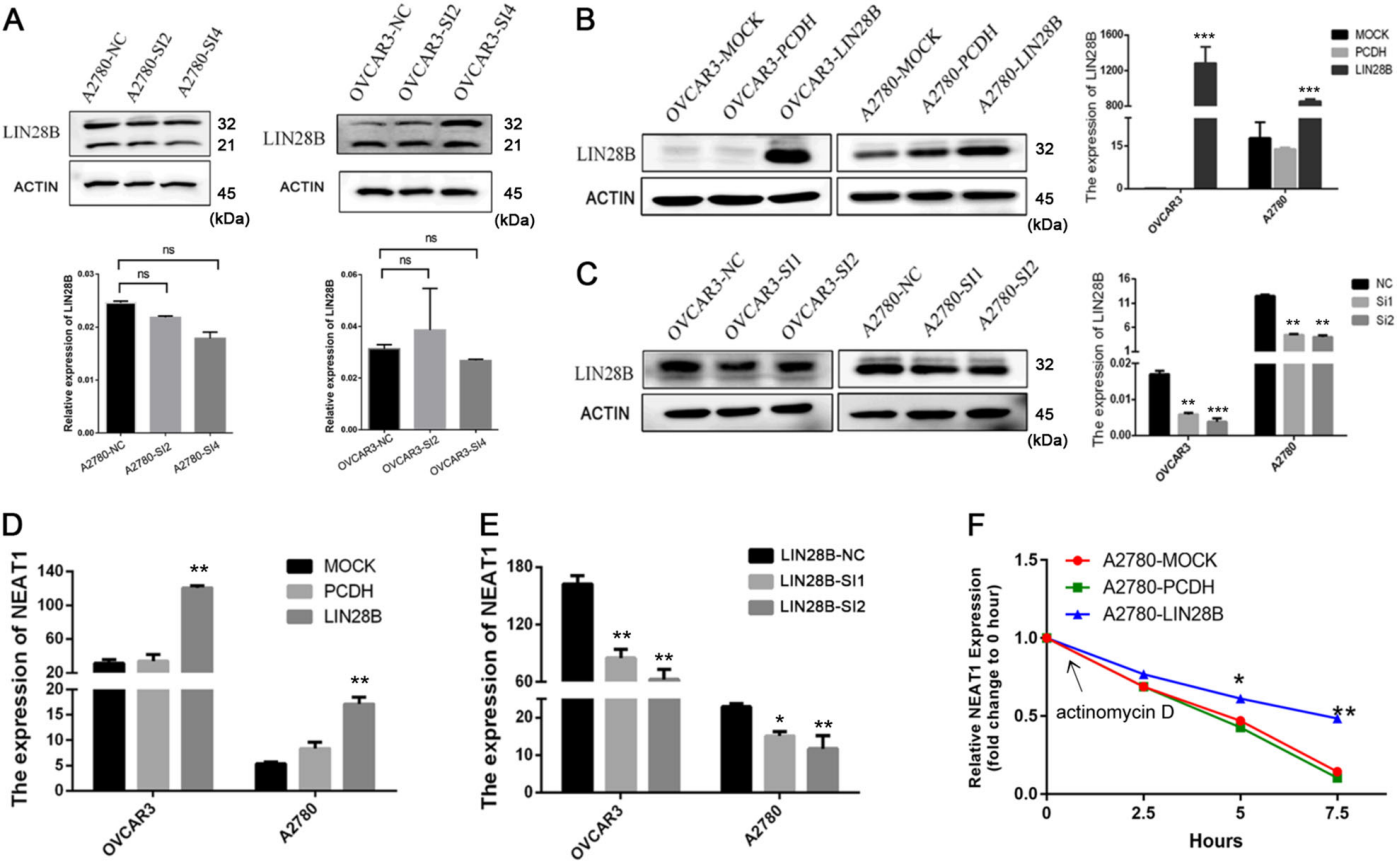
LIN28B enhances the stability of NEAT1. **a** Immunoblotting to evaluate the LIN28B protein levels after NEAT1 downregulation in OVCAR3 and A2780 cells. **b** The qRT-PCR analysis of LIN28B interference in EOC cells. **c** The EOC cell lines A2780 and OVCAR3 were transfected with either LIN28B or a vector control, and LIN28B overexpression was verified by qRT-PCR. Relative RNA levels of NEAT1 in OVCAR3 and A2780 cells with LIN28B knockdown (**d**) or overexpression (**e**) based on qPCR. **f** The half-life of NEAT1 after treatment with 2.5 μM actinomycin D for the indicated times with LIN28B overexpression in A2780 cells. ^*^P < 0.05, ^**^P < 0.01, and ^***^P < 0.001. PCDH represents empty control. ns not significant

Given that NEAT1 interacted with LIN28B in EOC cells, we characterized the molecular consequences of these associations. Interestingly, NEAT1 had no effect on the LIN28B protein levels or mRNA levels (Fig. 4a), prompting us to evaluate whether NEAT1 could be regulated by LIN28B in EOC cells. We evaluated the efficiency of overexpression or knockdown of LIN28B (PCDH: empty control; Fig. 4b, c). Overexpression of LIN28B enhanced the NEAT1 RNA levels, whereas silencing LIN28B decreased the NEAT1 RNA levels in A2780 cells (Fig. 4d, e). Moreover, actinomycin D, which effectively inhibits de novo RNA synthesis, was used to explore the stability of NEAT1. Overexpression of LIN28B resulted in increases in the NEAT1 half-life and RNA level (Fig. 4f), revealing that LIN28B specifically regulated NEAT1 stability in the EOC cells.

### NEAT1 is a functional downstream mediator of LIN28B

**Fig. 5:**
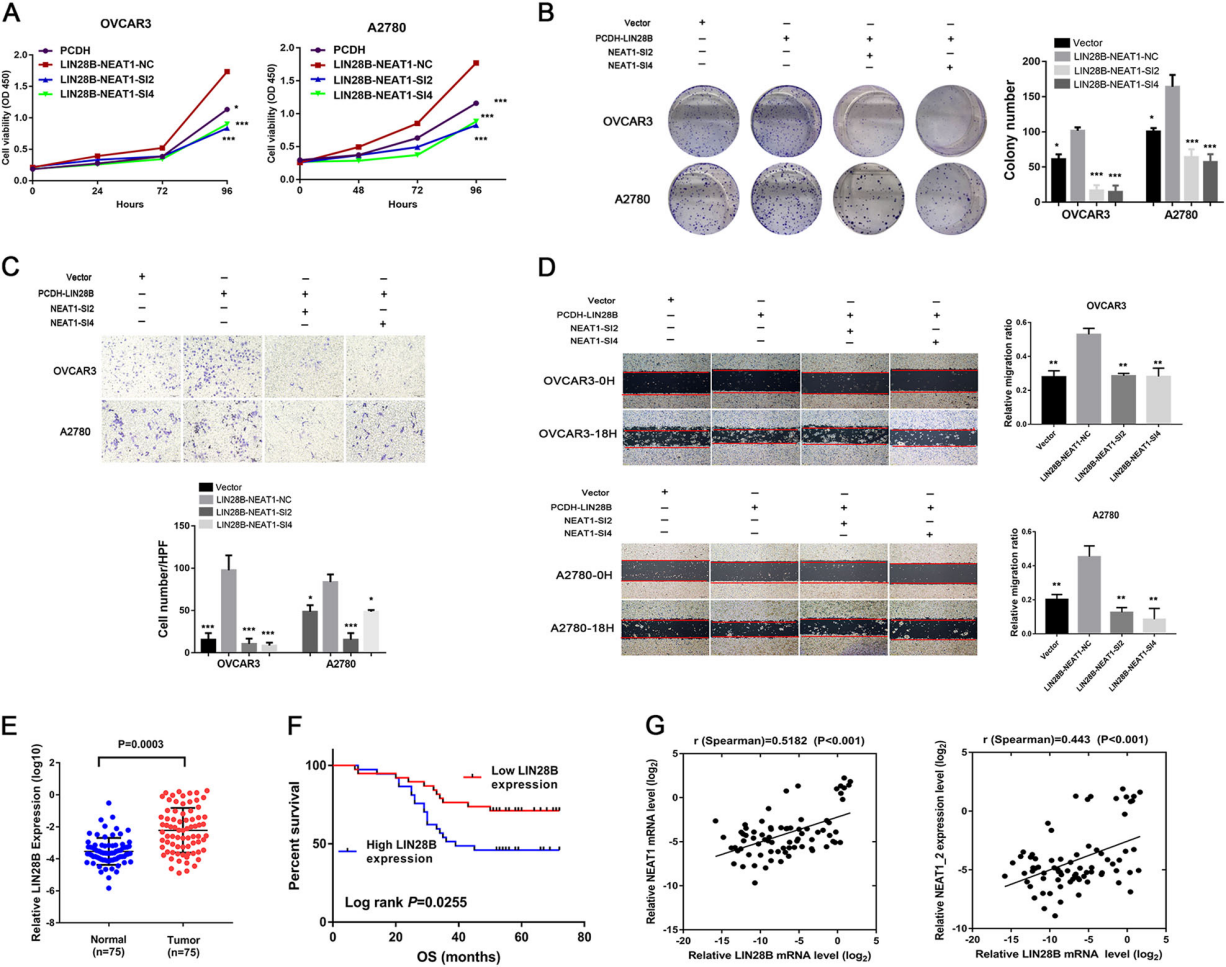
Rescue assays confirm that NEAT1 is an important downstream effector of LIN28B. **a** CCK-8 rescue assays were performed after NEAT1 knockdown in LIN28B-overexpressing cells. **b** The cell colony formation ability was used to evaluate cell growth after transfection with NEAT1-siRNA in LIN28B-overexpressing cells. **c** Transwell invasion rescue assays were performed after NEAT1 knockdown in LIN28B-overexpressing cells. Scale bar is 50 μm. **d** A wound-healing assay was applied to analyze the migratory capacity of EOC cells after transfection with the NEAT1-siRNA in LIN28B-overexpressing cells. **e** The differential expression of LIN28B in 75 HGSOC tissues and unpaired normal ovarian tissues was analyzed. **f** Kaplan–Meier analysis of OS of 75 HGSOC patients based on LIN28B mRNA expression was performed. The cut-off threshold used to divide the patients into high- and low-expression groups was the median value. **g** Pearson’s correlation curves are shown, revealing the positive relationship between LIN28B and either total NEAT1 or NEAT1-2. Scale bar is 200 μm. ^*^P < 0.05, ^**^P < 0.01, and ^***^P < 0.001

Considering the effect of LIN28B on NEAT1 stability, we hypothesized that the biological effects of NEAT1 may be partially regulated by LIN28B. We conducted in vitro experiments to investigate this hypothesis in ovarian cancer cells. The CCK-8 and colony formation assay results showed that LIN28B overexpression promoted OVCAR3 and A2780 cell proliferation, which was impaired by simultaneous knockdown of NEAT1 (Fig. 5a, b). The cell-wounding and Transwell assays also showed that NEAT1 knockdown partially attenuated the effects of LIN28B overexpression in ovarian cancer cells (Fig. 5c, d). Our results indicated that NEAT1 promoted ovarian cancer cell proliferation and metastasis partly in a LIN28B-regulated manner.

Considering the interaction between LIN28B and NEAT1 and the effect of LIN28B on NEAT1, we examined the LIN28B mRNA levels in HGSOC patients. Quantitative real-time PCR (qRT-PCR) revealed that LIN28B levels were much higher in HGSOC tissues than in normal ovarian tissues (Fig. 5e), and the survival analysis showed that high LIN28B expression was correlated with shorter OS (*P* = 0.0255, Fig. 5g) but had no significant correlation with PFS (*P* = 0.0794, Supplementary Figure S2). Moreover, Spearman’s correlation analysis suggested a significant positive correlation between LIN28B and each of total NEAT1 and NEAT1-2 in HGSOC tissues (*r* = 0.5182 and 0.443, *P* < 0.001, Fig. 5f), which further confirmed the relationship between LIN28B and NEAT1.

### NEAT1 knockdown inhibits the growth of HGSOC xenografts in vivo

**Fig. 6:**
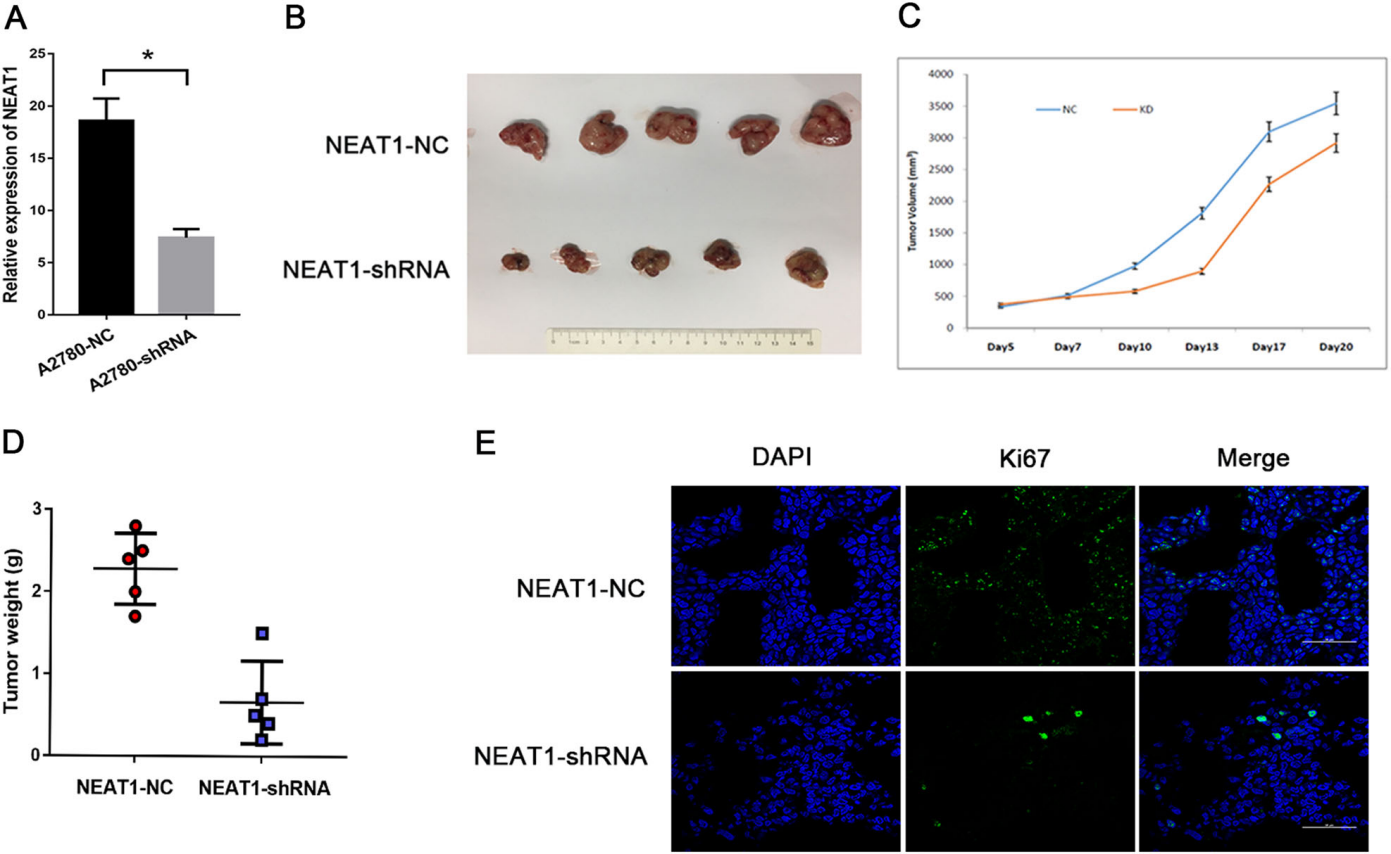
Knockdown of NEAT1 inhibits ovarian tumor growth in vivo. **a** NEAT1-knockdown stable cell lines were constructed, and the knockdown efficiency was tested by qRT-PCR. **b** Downregulation of NEAT1 expression attenuated tumor growth in nude mice. **c**, **d** The effect of NEAT1 on EOC tumor growth was evaluated based on the tumor volumes and tumor weights in the two groups. **e** An immunofluorescence assay was used to detect the Ki-67 expression levels in frozen sections. Scale bar is 50 μm. ^*^P < 0.05

To provide in vivo evidence for the oncogenic role of NEAT1 in HGSOC, first, we constructed NEAT1 knockdown stable cell lines; the knockdown efficiency is shown in Fig. 6a. Then, we used a xenograft mouse model. After subcutaneous injection of 10 mice with A2780-NC and A2780-shRNA cells, all of the mice developed detectable tumors. The growth of the xenograft was significantly inhibited in mice treated with the NEAT1 shRNA lentivirus compared with that in mice treated with the NC lentivirus. (Fig. 6b–d). To investigate the functions of NEAT1 in vivo, we used frozen mouse tissue sections to conduct an immunofluorescence assay. The results showed that Ki67, which is a proliferation index, had brighter fluorescence in the NEAT1-NC group than in the NEAT1-shRNA group (Fig. 6e). LIN28B expression of mice tumor tissues was detected by Western blot and immunofluorescence assay. The results revealed no differences in immunoblotting strips and fluorescence intensity between the NEAT1-NC group and the NEAT1-shRNA group (Supplementary Figure S3A, S3B), which provided strong support for the previous findings that NEAT1 had no effect on LIN28B.

### NEAT1 regulates tumor cell–cell adhesion via miR-506

**Fig. 7:**
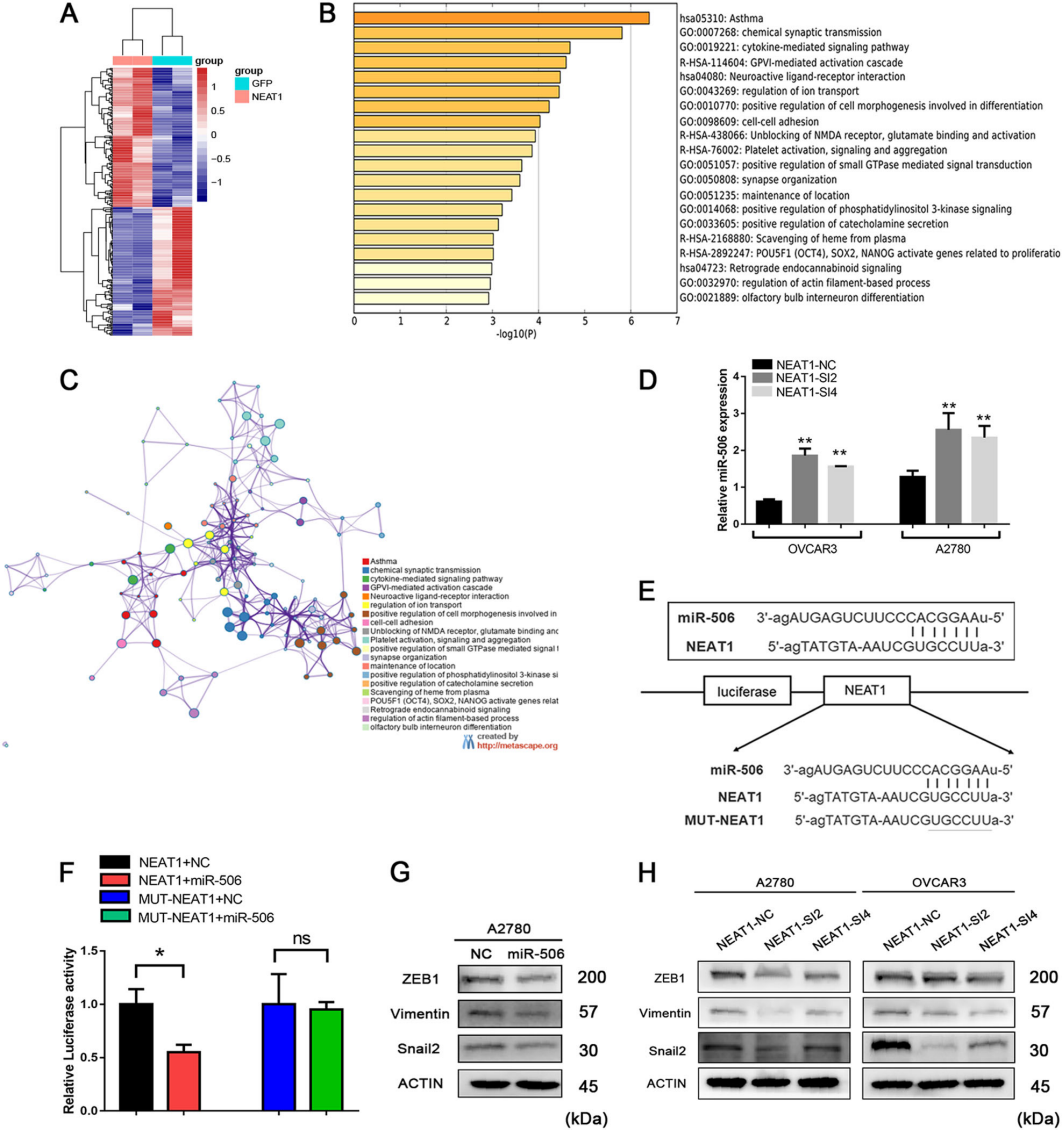
NEAT1 promotes cell proliferation and migration via miR-506. **a** Gene expression profiling was performed in NEAT1-knockdown HeLa cells. DEGs are presented in the form of a heat map. **b** DEGs were used to conduct an enrichment analysis. **c** A subset of representative enriched terms was converted into a network layout. **d** Relative miR-506 levels in OVCAR3 and A2780 cells with NEAT1 knockdown are shown. **e** The binding region between miR-506 and NEAT1 was predicted using bioinformatics analysis, and luciferase reporter plasmids containing the wild type (WT-NEAT1) or mutant NEAT1 (MUTNEAT1) sequence are shown. **f** WT-NEAT1 or MUT-NEAT1 was cotransfected into HEK-293T cells with miR-506 mimics or their corresponding negative controls; then, the luciferase reporter assay was performed. **g** Western blotting was used to detect the levels of miR-506 target genes, including ZEB1, Vimentin, and Snail2, following miR-506 overexpression in A2780 cells. **h** The levels of miR-506 target genes were detected by Western blotting when NEAT1 was downregulated in A2780 and OVCAR3 cells. ^*^P < 0.05 and ^**^P < 0.01. ns not significant

To identify the genes targeted by NEAT1 and to explore the mechanism of NEAT1 in tumorigenesis, gene expression profiling was performed on NEAT1-siRNA HeLa and control cells (GSE45158)^24^. A total of 387 differentially expressed genes (DEGs; >1-fold; false discovery rate = 0.05) were identified (Supplementary Table 2). A heat map of these DEGs is presented in Fig. 7a. Then, we conducted a gene annotation analysis through Metascape (http://metascape.org; Supplementary Table 3). The enrichment analysis showed that NEAT1 influenced multiple biological processes (Fig. 7b), including cell–cell adhesion, which plays pivotal roles in tumor cell proliferation and metastasis. We also selected a subset of representative terms from this cluster and converted them into a network layout (Fig. 7c).

Previous studies have shown that many lncRNAs function as ceRNAs for specific miRNAs^25^. To clarify the mechanism by which NEAT1 affects cell–cell adhesion, we focused on whether NEAT1 could act as a ceRNA. Target prediction tools (ChipBase and StarBase) and a literature review were applied to evaluate potential miRNAs. Among these miRNAs, miR-506 was chosen as the predicted candidate because (1) the expression of miR-506 was upregulated after NEAT1 knockdown (Fig. 7d), (2) it contained NEAT1 binding sites, and (3) it has a strong effect on the EMT^26,27^. The complementary binding sites between miR-506 and NEAT1 are shown in Fig. 7e. To validate whether miR-506 was a direct target of NEAT1, luciferase reporter plasmids expressing NEAT1 with wild type/mutant miR-506 binding sites were constructed in our study (Fig. 7e). Cotransfection of HEK-293T cells with the luciferase reporter plasmid containing the wild type binding sites and miR-506 mimics decreased the reporter activity relative to that in the negative control (Fig. 7f). Additionally, because miR-506 has been reported to target EMT-related genes, such as ZEB1, Vimentin, and Snail2, we examined whether NEAT1/miR-506 could influence these proteins. Overexpression of miR-506 downregulated ZEB1, Vimentin, and Snail2 in A2780 cells (Fig. 7g), and consistent results were found when NEAT1 was knocked down by siRNAs (Fig. 7h).

**Fig. 8:**
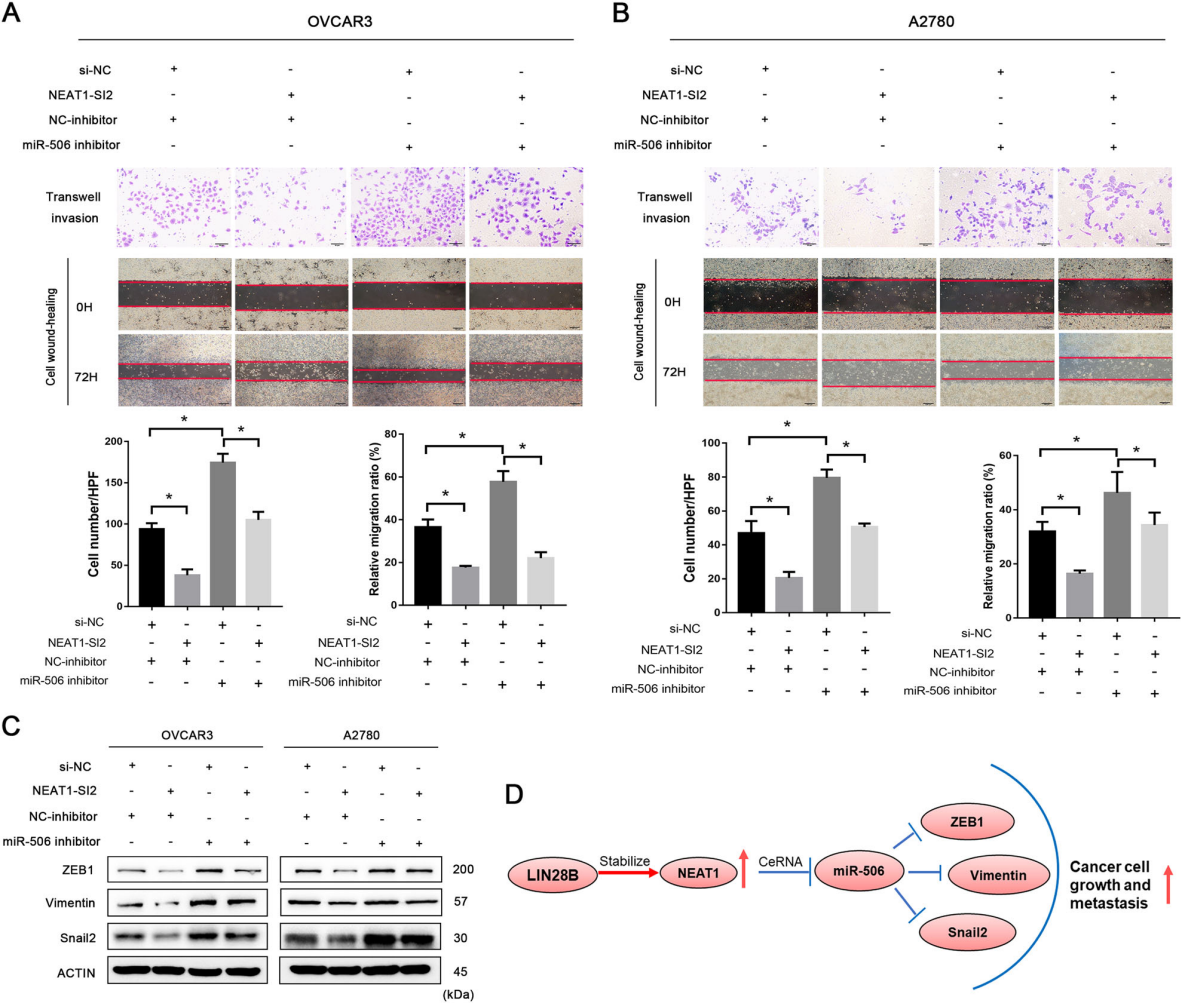
miR-506 reversed the promoting effect of NEAT1 on the cell migration capacity of ovarian cancer cells. **a**, **b** Transwell and cell wound-healing assays were conducted in OVCAR3 and A2780 cell lines cotransfected with NEAT1 siRNA and miR-506 inhibitor. Scale bars are 50 and 200 μm. **c** Western blotting was used to detect the levels of miR-506 target genes, including ZEB1, Vimentin, and Snail2, with NEAT1 siRNA and miR-506 inhibitor cotransfected in A2780 and OVCAR3 cells. **d** Integrated model depicting lncRNA NEAT1 as an oncogene in HGSOC. *P < 0.05

To further test our ceRNA hypothesis, rescue experiments were conducted. Knockdown of the endogenous highly expressed NEAT1 by NEAT1-SI2 significantly weakened the migration and invasion of OVCAR3 and A2780 cells (Fig. 8a, b). Suppression of miR-506 blocked the effects induced by NEAT1 depletion (Fig. 8a, b). Moreover, knocking down miR-506 alone greatly enhanced the invasive ability (Fig. 8a, b). The expression of the miR-506 targeted genes ZEB1, Vimentin, and Snail2 was analyzed. The influence of siNEAT1 on expression was reversed by miR-506 inhibitor in ovarian cancer cells (Fig. 8c). These results demonstrate that NEAT1 promotes tumor cell proliferation and metastasis according to the ceRNA patterns (Fig. 8d).

## Discussion

With the recent development of high-throughput techniques, a variety of systematic cancer genomics projects have been conducted to investigate different molecular pathways and to identify genomic, transcriptomic, and epigenomic alterations in cancers^28,29^. These investigations have focused not only on protein-coding genes but also on long noncoding transcripts, which have been shown to be involved in the regulation of a diverse array of biological processes. This study focused on NEAT1, which is a lncRNA that has been studied as the core structural component of paraspeckles^7,8,10^. A great deal of effort has been expended to understand the functional role of NEAT1 in cancer progression. For example, Zhen et al. showed that NEAT1 promoted glioma pathogenesis by interacting with the miR-449b-5p/c-Met axis^30^. In addition, NEAT1 was found to promote papillary thyroid cancer through regulating ATAD2 expression by sponging miR-106b-5p^31^. In regards to chemo-resistance, NEAT1 was found to upregulate EGCG-induced CTR1 and enhance cisplatin sensitivity in lung cancer cells^32^. Moreover, NEAT1 was found to be associated with malignant progression in ovarian cancer and was reported to have clinical significance^17,33,34^. However, elucidating its detailed involvement in ovarian tumorigenesis requires further investigation.

In our study, we found that the relative expression of NEAT1 was significantly increased in HGSOC tissues. High NEAT1 expression was correlated with tumor size. Additionally, NEAT1 was a potential independent prognostic factor for HGSOC. After knocking down NEAT1, the malignant behaviors of ovarian cancer cells were restrained. These results are consistent with those of the previous studies and support a role for NEAT1 as a functional oncogenic lncRNA in HGSOC.

Next, we sought to identify the underlying molecular mechanisms by which NEAT1 exerts its modulatory effect on malignant EOC phenotypes. Previous studies reported that posttranscriptional regulation through specific RBPs was emerging as a critical regulatory level for nearly all biological processes^19^. Recent reports have shown that NEAT1 interacts with some RBPs. Jiang et al. found that NEAT1 bound the RBPs NONO and PSF to enhance pre-miRNA processing^22^. To examine whether NEAT1 could bind to other RBPs in ovarian cancer cells, we performed a bioinformatics analysis, RNA pulldown assay, and RIP assay, which revealed that NEAT1 could combine with LIN28B.

The molecular mechanisms underlying the function of LIN28B, as a member of the RBP family, were initially suggested to predominantly include regulation of the let-7 miRNAs, which have essential functions in inflammation, wound healing, embryonic stem cells, and cancer^35–39^. LIN28B expression is associated with tumor initiation, progression, resistance, and poor outcomes in several solid cancers^40–43^, including ovarian cancer^44–46^. However, few reports have investigated the interactions between LIN28B and lncRNAs. Only lncRNA H19 has been found to interact with LIN28B^23^. Our results extend current knowledge on the functional role of lncRNA–LIN28 complexes. However, the longer transcript of NEAT1 has a weak binding capacity with LIN28B. There are a few reasons why the longer transcript NEAT1_2 has a much weaker binding capacity with LIN28B than does NEAT1_1. First, the two transcripts have different lengths, which may result in different secondary structures. Second, it is believed that NEAT1_2, not NEAT1_1, is indispensable for paraspeckle formation. Although they both promote tumorigenesis, these two NEAT1 isoforms are reported to be involved in different cellular activities^47^, suggesting that the binding capacity with LIN28B may differ between the two transcripts. Third, recent reports have shown that NEAT1_2 interacts with some RBPs^48^. It is possible that other RBPs may affect the combination of LIN28B and NEAT1_2. Fourth, primers are responsible for different binding capacities. In the present study, we designed two primers named NEAT1 (which can detect both transcripts) and NEAT1-2 (which can only detect the long transcript), so the abundance of NEAT1 in cells differed between the two primers used for detection. The present study identified LIN28B as an important player in the regulation of NEAT1 expression by enhancing its stability. Rescue assays confirmed the function of the LIN28B/NEAT1 axis in ovarian cancer cells.

Additional findings of the present study involve the identification of downstream molecules associated with NEAT1 functions. The data obtained from the GEO database (GEO accession: GSE45158) indicated that NEAT1 influences multiple biological processes, including cell–cell adhesion. Cell adhesion plays a pivotal role in tumorigenesis and is regarded as an important component of the EMT. Emerging evidence suggests that lncRNAs may function as ceRNAs or molecular sponges to modulate the activities of miRNAs. To further elaborate the relationship between NEAT1 and EMT, we investigated whether NEAT1 could act as a ceRNA. As a recognized EMT-associated molecule, miR-506 has been reported to be expressed in many types of cancer and to regulate EMT-associated genes, such as Snail2, Vimentin, and ETS-1^27,49,50^. Luciferase assays and Western blotting showed that NEAT1 might sponge miR-506 to promote tumorigenesis. Future work is needed to elucidate the detailed mechanism by which NEAT1 regulates miR-506 and to further delineate the network controlled by NEAT1 during HGSOC progression.

In summary, this study is the first report to investigate a potential interaction between NEAT1 and LIN28B during HGSOC progression. We found that NEAT1 was overexpressed and was associated with poor patient survival. NEAT1 promoted EOC cell proliferation, tumor invasion and metastasis in vitro, and cell proliferation in vivo. Moreover, we observed that NEAT1 might promote tumorigenesis by sponging miR-506. Our findings provide new insights into the molecular details of HGSOC and potential therapeutic targets to combat this disease.

## Materials and methods

### Human samples and tissue handling

The study was undertaken with the understanding and written consent of each subject. The study methodologies conformed to the standards set by the Declaration of Helsinki. All human tissues were collected using the protocols approved by the Human Ethics Committee of the Fudan University Shanghai Cancer Center. HGSOC tissues were obtained from 75 patients who underwent surgical resection of ovarian cancer between 2009 and 2012. Normal ovarian tissues were collected from cervical cancer surgery patients. No local or systemic treatment was administered to these patients prior to the operation. All tissue samples were washed with sterile phosphate-buffered saline before being snap frozen in liquid nitrogen and stored at −80 °C until analysis. The pathological parameters were appraised by an experienced pathologist. Patient follow-up was performed every 3 months during the first year postsurgery and then every 3–6 months thereafter until April 1, 2018. Disease-free survival (DFS) was calculated from the date of surgery to the date of recurrence or the last follow-up as appropriate.

### RNA isolation, reverse transcription, and quantitative real-time PCR

Total RNA was extracted from the tissue samples and cell lines using TRIzol reagent (Invitrogen, Carlsbad, CA, USA) according to the manufacturer’s protocol. Reverse transcription (RT) and qRT-PCR kits (Takara, Dalian, China) were utilized to evaluate the mRNA expression levels of the indicated genes. PCR primers were designed and synthesized using a primer design tool (http://www.ncbi.nlm.nih.gov/tools/primer-blast/); the primer sequences are listed in Supplementary Table 4. The relative quantification value for each target gene was expressed as 2^−ΔΔCT^. β-Actin was used as an internal reference for the mRNAs, and U6 served as an internal reference for the miRNAs.

### Cell culture and treatments

HEK-293T cells and the human EOC cell lines A2780, HO8910, SKOV3, OVCAR3, CAOV3, ES-2, and OV420 were cultured in Dulbecco’s modified Eagle’s medium (Gibco, Carlsbad, CA, USA) supplemented with 10% fetal bovine serum (FBS) (Gibco), 50 U/mL of penicillin and 50 µg/mL of streptomycin (Gibco). All cell lines were maintained at 37 °C and 5% CO_2_ in a humidified atmosphere.

### Lentivirus, siRNA, miR-506 inhibitor, and transfection

The NEAT1-shRNA-Lentivirus for use in the in vivo experiments was purchased from the ABM Company. LIN28B-related stable cell lines were constructed according to previously published guidelines^51^. siRNAs targeting NEAT1 and LIN28B were purchased from Suzhou Synbio Technologies and transfected into cells using Lipofectamine 3000 (Invitrogen). All siRNA and shRNA sequences are provided in Supplementary Table 5. miR-506 inhibitor was purchased from GenePharma (Shanghai, China).

### Cell proliferation assays

Cells (2 × 10^3^ per well) were seeded into 96-well plates 24 h before the experiment. A2780 and OVCAR3 cells were transfected with siRNAs or a scramble siRNA. Proliferation was measured using the CCK-8 kit (Dojindo, Japan) according to the manufacturer’s protocol. All experiments were performed in triplicate. The cell proliferation curves were plotted using the absorbance at each time point.

### Colony formation assay

Cells were resuspended into single-cell suspensions 48 h posttransfection. For the colony formation assay, 1000 cells were plated into 6-well plates and incubated in the corresponding medium with 10% FBS at 37 °C. After 2 weeks, the cells were fixed and stained with 0.1% crystal violet. The visible colonies were manually counted. Triplicate wells were measured for each treatment group.

### Cell wound-healing and invasion assays

For the wound-healing assay, cells were seeded into 6-well plates and allowed to grow to 90–95% confluence. A single scratch wound was created 6 h after transfection with the siRNAs. The cells were washed with PBS to remove cell debris, supplemented with serum-free medium, and monitored. Images were captured by phase-contrast microscopy at 0, 18, 24, and 36 h after wounding.

A cell invasion assay was performed using Transwell chamber inserts (8.0 mm, Corning, NY, USA) in a 24-well plate. Then, 2 × 10^4^ cells suspended in 200 µL of serum-free medium were added to the upper chamber. Culture medium containing 20% FBS was placed in the bottom chamber. The cells were incubated for 24 or 48 h at 37 °C. After incubation, the cells on the upper surface were scraped and washed away, whereas the cells on the lower surface were fixed with 20% methanol and stained with 0.1% crystal violet. The numbers of invaded cells were counted in five randomly selected fields under a microscope. The experiments were repeated independently in triplicate.

### RNA pulldown assays

Due to the long length of NEAT1, we designed two probes (wild type and mutant) that we labeled using the Biotin RNA Labeling Mix (Thermo Fisher, USA), treated with RNase-free DNase I (Takara, Japan) and purified with the RNeasy Mini Kit (QIAGEN, USA). Next, 1 pmol of biotinylated RNA was pretreated with RNA structure buffer (Beyotime Biotechnology, Shanghai, China) to ensure appropriate secondary structure formation. The pretreated biotinylated RNAs were incubated with 1 mg of protein extract from the A2780 cells at 4 °C for 4 h, gently mixed with 40 μL of washed streptavidin beads (Invitrogen, CA, USA) and incubated on a rotator overnight according to the manufacturer’s protocol (Thermo Fisher, Waltham, USA). The proteins were precipitated and diluted in 60 μL of protein lysis buffer and then detected by Western blotting.

### RBP immunoprecipitation and RIP-qPCR assays

We performed RIP experiments using a Magna RIP RNA-Binding Protein Immunoprecipitation Kit (Millipore, Billerica, MA, USA) according to the manufacturer’s instructions. A rabbit anti-LIN28B antibody (1:50; Cell Signaling Technology) was used for the experiments. The lysates were incubated with the antibody overnight at 4 °C. The coprecipitated RNAs were detected by real-time PCR. The corresponding primers are provided in Supplementary Table 4.

### Western blotting assay

Cells were lysed in RIPA buffer (Sigma-Aldrich) supplemented with a protease inhibitor (Roche, Basel, Switzerland) and a phosphatase inhibitor (Roche). The protein concentration was measured using a BCA protein assay kit (Thermo Scientific, USA). The primary rabbit anti-LIN28B, anti-MMP2, anti-MMP9, anti-N-Cadherin, and anti-E-Cadherin antibodies were purchased from Cell Signaling Technology. The rabbit anti-ZEB1, anti-vimentin, and anti-Snail2 antibodies were purchased from Proteintech.

### Mouse xenografts and immunofluorescence

Female BALB/c nude mice 4–5 weeks of age were purchased from the Shanghai Laboratory Animal Center at the Chinese Academy of Sciences. All experiments were performed in accordance with relevant institutional and national guidelines and the regulations of the Shanghai Medical Experimental Animal Care Commission. Mice (5 per group) were injected subcutaneously with 0.2 mL of a cell suspension containing 5 × 10^5^ cells (the piLenti-shRNA-VECTOR and piLenti-shRNA-NEAT1 stable A2780 cell lines) in the right axilla. The tumor growth rates were monitored. When a tumor was palpable, it was measured every other day, and its volume was calculated according to the formula: volume = length × width^2^ × 0.5.

To investigate the relationship between NEAT1 and LIN28B in vivo, frozen sections from animal experimental tumors were washed with PBS and fixed with 4% paraformaldehyde for 20 min at room temperature. After three washes with PBS, the sections were blocked in 5% goat serum for 1 h. The cells were subsequently incubated with primary antibodies specific for Ki-67 (1:100, Proteintech) and LIN28B (1:50, Abcam; ab71415) overnight at 4 °C. The next day, the sections were washed with PBS and then incubated with fluorescence-conjugated secondary antibodies (Beyotime, China), followed by DAPI. Images were captured under a fluorescence microscope.

### Luciferase assay

The wild type (WT) or mutant (MUT) NEAT1-binding sites were subcloned into the pGL3 Basic vector (Promega, Madison, WI, USA). HEK-293T cells were seeded onto 96-well plates. Mimics of miR-506 or an NC (RiboBio, Guangzhou, China) were cotransfected with pLUC-WT-NEAT1 or pLUC-MUT-NEAT1. Two days after transfection, the cells were collected, and the luciferase activity was determined using the Dual Luciferase Reporter Assay System (Promega, Madison, WI, USA).

### GEO dataset analysis

The GSE45158 dataset acquired from the GEO database was analyzed using GEO2R (https://www.ncbi.nlm.nih.gov/geo/geo2r/) to identify DEGs following NEAT1 downregulation. Genes with *P* values < 0.05 and |logFC| > 1 were considered DEGs. In total, 387 DEGs were identified. Then, gene annotation analysis was conducted with Metascape (http://metascape.org).

### Statistical analysis

All statistical analyses were performed using SPSS 18.0 (IBM, SPSS, Chicago, IL, USA). The significance of differences between groups was estimated using Student’s *t*-test, the *χ*^2^ test, or the Wilcoxon test as appropriate. The OS and DFS rates were calculated using the Kaplan–Meier method with the log-rank test for comparison. The survival data were evaluated using univariate and multivariate Cox regression analyses. A value of *P* < 0.05 indicated a significant difference.

## Electronic supplementary material

Supplementary Figures

Supplementary Table1

supplementary Table2

supplementary Table3

Supplementary Table4

Supplementary Table5
