# Supplementary material for: Long noncoding RNA NEAT1, regulated by LIN28B, promotes cell proliferation and migration through sponging miR-506 in high-grade serous ovarian cancer
Source: Cell Death Dis. 2018 Aug 28;9(9):861. doi: 10.1038/s41419-018-0908-z (PMC6113267; doi:10.1038/s41419-018-0908-z)
Supplement: Supplementary file 1 — Supplementary Figures [file 41419_2018_908_MOESM1_ESM.docx]

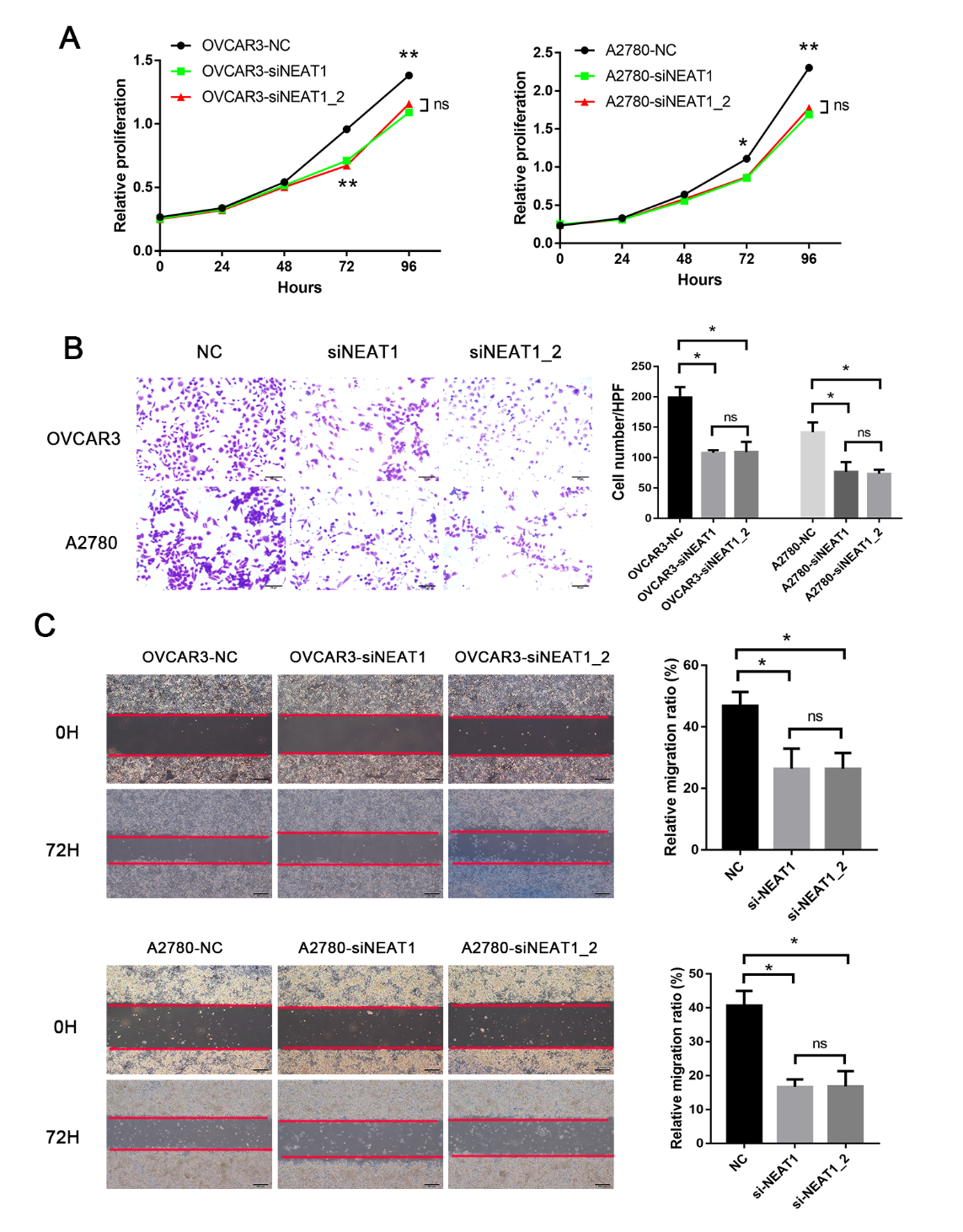


**Supplementary Figure S1** siRNAs targeting both NEAT1 transcripts or NEAT1_2 resulted in nearly identical arrest of ovarian cancer cell proliferation and migration. (**A**) CCK-8 assays were performed to determine the difference in proliferation between these two transcripts. (**B**) The cell invasion potential was assessed following targeting of the different transcripts using a Transwell assay. Scale bar is 50 μm. (**C**) The cell migration ability following targeting of the different transcripts was evaluated using a wound-healing assay; images of OVCAR3 and A2780 cells were taken at 0 and 72 h postscratch. Scale bar is 200 μm. ^*^*P<*0.05.


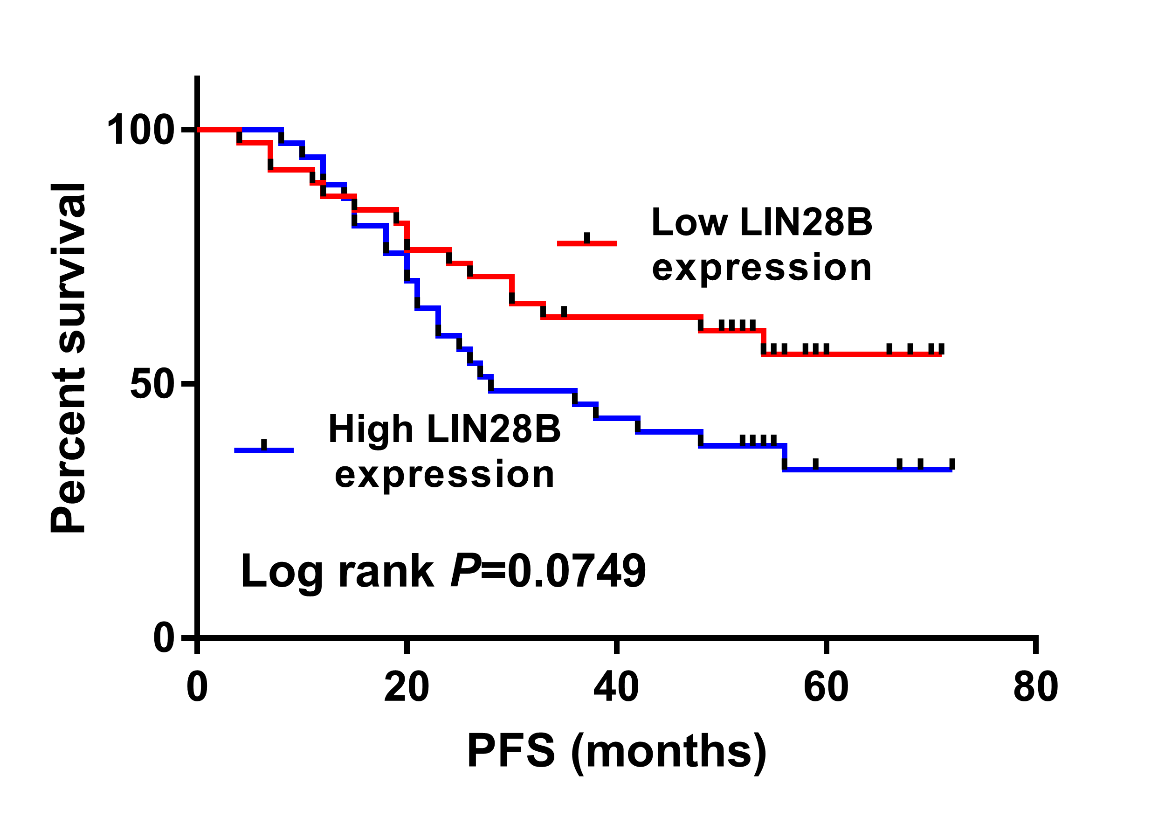


**Supplementary Figure S2** High LIN28B mRNA expression tended to be associated with shorter PFS, but the relationship was not statistically significant (P=0.0794).


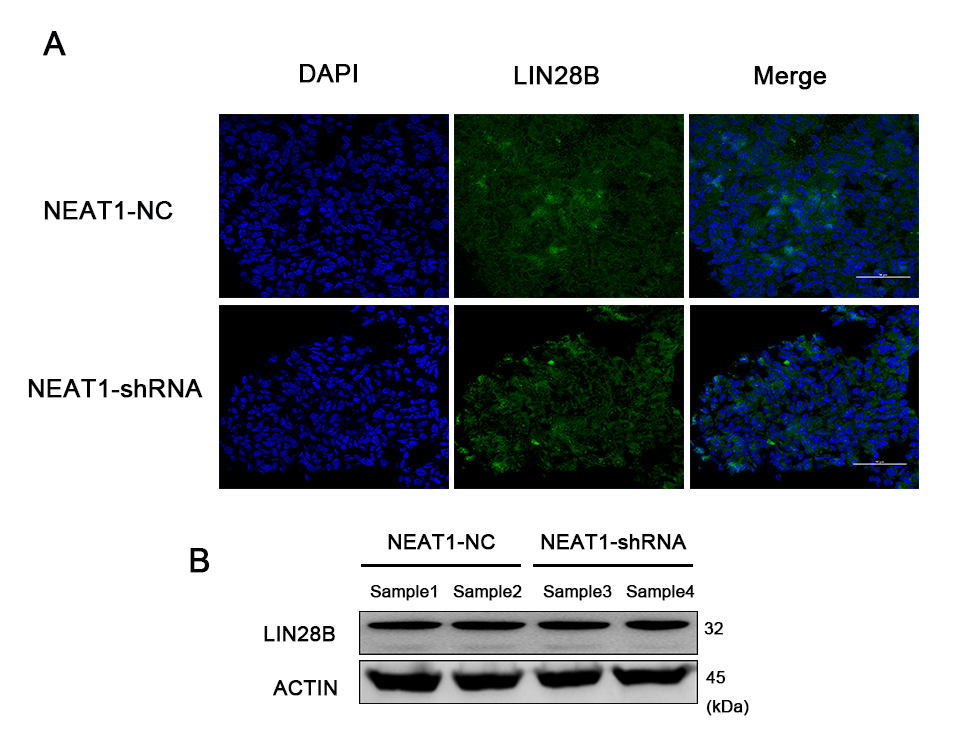


**Supplementary Figure S3** Fluorescence intensity and immunoblotting strips of LIN28B of mice tumor tissues revealed no differences between the NEAT1-NC group and NEAT1-shRNA group. (**A**) LIN28B expression of mice tumor tissues was detected by immunofluorescence assay. Scale bar is 50 μm. (**B**) Mice tumor tissues were ground, and the protein was extracted; then, LIN28B protein levels were analyzed by Western blotting assay.
